# The complete mitochondrial genome of *Tanacetipathes thamnea* Warner, 1981 (Antipatharia: Myriopathidae)

**DOI:** 10.1080/23802359.2019.1692701

**Published:** 2019-11-20

**Authors:** Diego Francisco Figueroa, David Hicks, Nicole Jewel Figueroa

**Affiliations:** School of Earth, Environmental, and Marine Sciences, University of Texas Rio Grande Valley, One West University Boulevard, Brownsville, TX, USA

**Keywords:** Black coral, mitogenome, phylogeny, Gulf of Mexico

## Abstract

Specimens of the black coral *Tanacetipathes thamnea* were collected from the Northwestern Gulf of Mexico. The complete mitochondrial genome of one of these specimens was obtained from genomic DNA by next-generation sequencing technology on the Illumina HiSeq 2500. Only three species of black corals have a completely sequenced mitochondrial genome. These were used to reconstruct the phylogeny for the order Antipatharia. The mitochondrial genome of *T. thamnea* is 17,712 base pairs and contains 13 protein-coding genes, 2 ribosomal RNAs, and 2 transfer RNAs in the following order: 16s RNA, *COX3*, *COX1* (with intron), *ND4L*, *COX2*, *ND4*, *ND6*, *ATP8*, *ATP6*, and *ND5* (with intron and copies of *ND1* and *ND3*), *tRNA-Trp*, *ND2*, *12s RNA*, *CYTB*, *tRNA-Met*. The gene arrangement is the same as that for *Myriopathes japonica* with a nearly identical sequence (99.35% identical). These results show that the mitochondrial genome within the family Myriopathidae is highly conserved.

There are only three species of black corals (order Antipatharia) with completely sequenced mitochondrial genomes, *Myriopathes japonica*, *Chrysopathes formosa*, and *Stichopathes lutkeni* (Brugler and France 2007; Kayal et al. 2013). We present the first complete mitochondrial genome for the genus *Tanacetipathes* (GenBank: MN265369). The genus *Tanacetipathes* has 11 species (WoRMS Editorial Board 2019). *Tanacetipathes thamnea* is distributed throughout the western Atlantic, including the Gulf of Mexico (WoRMS Editorial Board 2019).

Specimens were collected at Aransas Bank (latitude 27° 35′ N and longitude 96° 27′ W) off the coast of Texas in the Gulf of Mexico and preserved in 95% ethanol. An individual polyp was picked from one specimen (UTRGV Coastal Studies Laboratory accession #D08-28B) and genomic DNA was extracted using the PureLink Genomic DNA Kit (Thermo Fisher Scientific Co., Waltham, MA). An indexed DNA library was prepared with the Nextera X2 kit (Illumina). This library was multiplexed with 95 other indexed libraries and sequenced on a 100 bp paired-end lane of Illumina HiSeq 2500 (Illumina, San Diego, CA) at Harvard’s Biopolymers facility. Reads were assembled de novo using CLC Genomics Workbench version 11 (Qiagen). The contigs from the assembly included the full mitochondrial genome with an average coverage of 54, which was then annotated using the mitochondrial genome for *M. japonica* (GenBank: JX456459) as a reference.

The mitochondrial genome of *T. thamnea* is 17,712 base pairs. It contains 13 protein-coding genes, 2 ribosomal RNAs, and 2 transfer RNAs, all in the major strand in the following order: *16s RNA*, *COX3*, *COX1* (with intron), *ND4L*, *COX2*, *ND4*, *ND6*, *ATP8*, *ATP6*, *ND5* (with intron and copies of *ND1* and *ND3*), *tRNA-Trp*, *ND2*, *12s RNA*, *CYTB*, and *tRNA-Met*. The nucleotide composition is 26.5% A, 18.2% C, 22.0% G, and 33.4% T (40.2% C + G and 59.8% A + T).

A concatenated alignment was created with the mitochondrial genomes of *M. japonica* (GenBank: JX456459), *Chrysopathes formosa* (GenBank: DQ304771), *Stichopathes lutkeni* (GenBank: JX023266), and *Metridium senile* (GenBank: HG423143) by extracting and aligning individual genes and RNAs using MUSCLE version 3.8 (Edgar 2004). Phylogenetic analyses were performed by maximum likelihood using Mega-X (Kumar et al. 2018) with bootstrap values from 1000 replicates and evolutionary model GTR + G+I. The resulting phylogeny shows that *T. thamnea* is most closely related to *M. japonica* (Figure 1). Both of these species belong to the family Myriopathidae. Sister to this clade is *Chrysopathes formosa* from the family Cladopathidae while *Stichopathes lutkeni*, from the family Antipathidae, is in the basal position. The mitochondrial genome within the family Myriopathidae is highly conserved. The mitochondrial genomes of *T. thamnea* and *M. japonica* are 99.35% identical.

**Figure 1.**
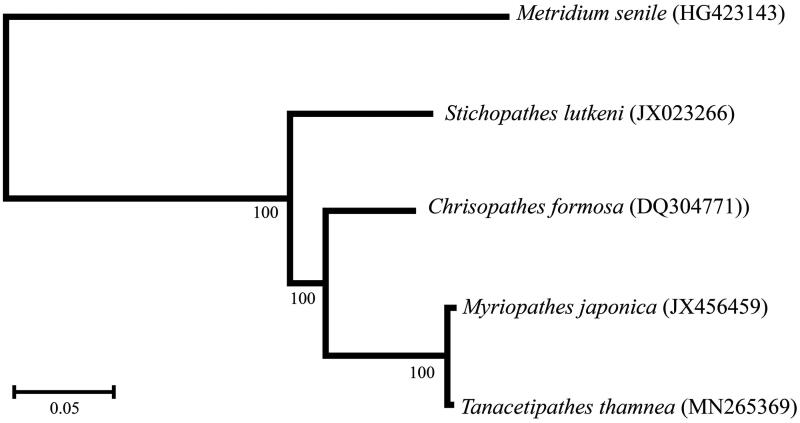
Black coral (Antipatharia) maximum-likelihood tree based on complete mitochondrial genomes. The anemone *Metridium senile* is used as the outgroup. Branch labels show bootstrap support.

The rate of evolution of the mitochondrial genome in this family is very low and it rivals the slow rate found within Octocorals, where mitogenomes of congeners only vary by 0.3–3.0% (e.g. Figueroa and Baco 2014). In Octocorals, this can be explained by a mitochondrial repair gene (*MutS*) found within their mitogenome (e.g. McFadden et al. 2006). A similar repair gene has not been found in Antipatharians, therefore, the reason for the slow rate of evolution of the mitochondrial genome within the family Myriopathidae remains to be explained.
